# First Report of Polymorphisms and Genetic Characteristics of *Prion-like* Protein Gene (*PRND*) in Cats

**DOI:** 10.3390/ani14233438

**Published:** 2024-11-27

**Authors:** Min-Ju Jeong, Yong-Chan Kim, Byung-Hoon Jeong

**Affiliations:** 1 Korea Zoonosis Research Institute, Jeonbuk National University, 820-120 Hana-ro, Iksan 54531, Republic of Korea; minju5149@jbnu.ac.kr; 2 Department of Bioactive Material Sciences, Jeonbuk National University, Jeonju 54896, Republic of Korea; 3 Department of Biological Sciences, Andong National University, Andong 36729, Republic of Korea; kych@anu.ac.kr

**Keywords:** prion disease, cats, *prion-like* protein gene, *PRND*, polymorphism, SNP, doppel

## Abstract

Prion diseases are fatal neurodegenerative disorders caused by the misfolding of the normal cellular prion protein (PrP^C^) into its infectious isoform (PrP^Sc^). Although prion diseases in humans, sheep, goats, and cattle have been extensively studied, feline spongiform encephalopathy (FSE) remains poorly understood. Genetic factors, particularly polymorphisms in the prion protein gene (*PRNP*) and *prion-like* protein gene (*PRND*), have been linked to prion disease susceptibility in various species. However, no studies have yet investigated the *PRND* gene in cats with respect to prion diseases. Therefore, we investigated polymorphisms in the feline *PRND gene* and analyzed their genetic characteristics. We sequenced the coding region of the *PRND* gene using samples from 210 domestic cats and determined the genotype and allele frequencies of *PRND* polymorphisms. We identified thirteen novel single nucleotide polymorphisms (SNPs), including six non-synonymous variants and one insertion/deletion (InDel) in the feline *PRND* gene. Four of the non-synonymous SNPs were predicted to have deleterious effects on the Doppel protein’s structure and function. Notably, the SNP c.97A>G (I33V) showed potential structural clashes, and the others formed additional hydrogen bonds. The LD analysis revealed strong genetic associations between the *PRND* SNPs and the *PRNP* InDel, suggesting linkage between these loci in cats. This study identifies novel *PRND* polymorphisms in domestic cats and provides new insights into the genetic factors underlying feline susceptibility to prion diseases. The strong genetic linkage between *PRND* and *PRNP* polymorphisms, coupled with predictions of detrimental effects on Doppel protein structure, suggests that *PRND* gene variants could influence prion disease progression in cats. These findings provide a foundational framework for future studies on the functional implications of *PRND* polymorphisms in FSE. To the best of our knowledge, this study is the first report on the genetic characteristics of *PRND* polymorphisms in cats.

## Simple Summary

Cats are important subjects in the study of feline spongiform encephalopathy (FSE), but the genetic factors contributing to their susceptibility have yet to be identified. Since the *prion-like* protein gene (*PRND*) is known to play a significant role in prion disease susceptibility among the prion protein gene family, investigating the genetic characteristics of the *PRND* gene in cats is essential. In this study, we identified 13 novel genetic variations. Using in silico tools, we found that four non-synonymous single nucleotide polymorphisms (SNPs)—c.76G>A (A26T), c.97A>G (I33V), c.251A>G (Q84R), and c.469C>A (L157I)—have the potential to disrupt protein structure and function. Linkage analysis revealed strong associations between *PRND* SNPs c.97A>G and c.251A>G and the *PRNP* InDel c.214_240delCCCCACGCCGGCGGAGGCTGGGGTCAG (p.76_84delPHAGGGWGQ), suggesting a shared genetic influence on disease susceptibility. This is the first study to investigate the genetic characteristics of the *PRND* gene in cats, and this analysis is expected to provide valuable baseline data for future functional studies.

## 1. Introduction

Prion diseases are a group of lethal neurodegenerative disorders caused by the misfolding of the normal cellular prion protein (PrP^C^) into its pathogenic isoform (PrP^Sc^) [1]. These misfolded proteins are infectious and can induce further misfolding of PrP^C^, leading to a cascade of neuronal damage [2]. Prion diseases have been reported in various host species, including Creutzfeldt-Jakob disease (CJD) in humans, scrapie in sheep and goats, bovine spongiform encephalopathy (BSE) in cattle, chronic wasting disease in cervids, and feline spongiform encephalopathy (FSE) in felines [3,4].

The first case of FSE, reported in 1990, coincided with the BSE outbreak [5]. Although other animal species that were presumably exposed to similar contaminated products did not show prion infection [6,7], various feline animals demonstrated the transmission of FSE [8,9,10,11,12]. Interestingly, FSE exhibits immunohistochemical and biochemical properties similar to those of BSE, suggesting that FSE might have originated from BSE [13,14,15]. However, since the mechanisms underlying prion disease pathogenesis are not yet fully understood, the factors contributing to the occurrence of FSE in felid species need to be investigated.

In previous studies, polymorphisms in the prion protein gene (*PRNP*) have been well established as a factor influencing susceptibility to prion diseases [16,17,18,19]. In humans, the Met/Met genotype at codon 129 is strongly associated with significantly increased risk for both sporadic and variant Creutzfeldt-Jakob disease (CJD) [20,21,22]. In sheep, haplotypes defined by codons 136, 154, and 171 are categorized into five risk levels, each linked to varying degrees of susceptibility to scrapie [16]. Additionally, single nucleotide polymorphisms (SNPs) at codons 102, 127, 142, 143, 146, 154, 211, and 222 in the caprine *PRNP* gene are key genetic markers used to predict susceptibility to scrapie in goats, with specific genotypes providing greater resistance [17,18,23,24,25,26,27,28,29,30,31,32,33,34,35].

The study of these polymorphisms has been extensively reported on in prion protein gene families across various species [36,37,38,39,40,41,42,43]. The family of prion protein genes includes *PRNP*, the *prion-like* protein gene (*PRND*), the prion-related protein gene (*PRNT*), and the shadow of prion protein gene (*SPRN*) [3,19]. Among these, *PRND* is particularly notable for its close proximity to *PRNP* and the biochemical and structural similarities between the *prion-like* protein (Doppel) and PrP [44,45]. Moreover, SNPs at codon 174 and 3′ UTR +28 in the human *PRND* gene differ significantly between sporadic CJD patients and the healthy population [46,47]. In cattle, SNPs at codons 95 and 132 of the bovine *PRND* gene are associated with the BSE-affected population [48]. In sheep, the GG genotype of SNP c.78G>A (A26A) in the ovine *PRND* gene is significantly associated with the ARR/ARR genotype of the *PRNP* gene, which is known to confer resistance in the scrapie risk group, which is composed of codons 136, 154, and 171 [49]. Additionally, SNPs c.28T>C, c.151A>G, and c.385G>C in the caprine *PRND* gene are strongly linked with the SNP c.428A>G (H143R) of the *PRNP* gene, which is associated with scrapie progression [50].

To date, no studies have investigated *PRND* polymorphisms in cats in relation to prion diseases. However, cats are considered to be a prion disease-sensitive species, and the various genetic factors that might influence disease susceptibility need to be investigated. Therefore, we analyzed polymorphisms of the *PRND* gene in cats and here report their genetic characteristics. We examined the genotype and allele frequencies of *PRND* polymorphisms in a sample of 210 domestic cats. Additionally, we used in silico prediction tools and 3D structural modeling to evaluate the impact of non-synonymous SNPs on the structure and function of the Doppel. We also conducted a linkage disequilibrium (LD) analysis to identify genetic associations between *PRNP* and *PRND* SNPs.

## 2. Materials and Methods

### 2.1. Sample Preparation

All 210 cat samples were provided by Hemalgeun and Cool-Pet veterinary hospitals (Anyang, Republic of Korea). These samples consisted of blood and tissue specimens that were obtained as byproducts from neutering surgeries or health check-ups performed at the veterinary hospitals [42,43]. Since these blood samples were obtained from cats owned by different individuals during health checkups, there is no blood relationship. Genomic DNA was isolated from the blood and tissue specimens by following the manufacturer’s manuals for a Bead genomic DNA prep kit for blood (Biofact, Daejeon, Republic of Korea) and a Labopass tissue genomic DNA isolation kit (Cosmogenetech, Seoul, Republic of Korea). All experimental procedures were approved by the Jeonbuk National University Institutional Animal Care and Use Committee (IACUC Number: CBNU 2019-00077). The procedures followed the guidelines set by the Korea Experimental Animal Protection Act.

### 2.2. Genetic Analysis of the Feline PRND Gene

Previous studies have suggested that *PRND* polymorphisms potentially associated with prion diseases are predominantly located near the open reading frame (ORF) of the PRND gene [46,47,48,49,50,51]. To explore the distribution of polymorphisms in this region, we designed a primer pair (GTACAACCGGAGCATGGGAA and CTCAGTACCTTCGGGACACG) targeting the ORF and its flanking sequences, based on the PRND sequence of Felis catus (Gene ID: 101087569) in GenBank from the National Center for Biotechnology Information. Polymerase chain reaction (PCR) was performed using an S-1000 thermal cycler (Bio-Rad, Hercules, CA, USA). The annealing temperature for the PCR experiment was optimized to 61 °C, to the manufacturer’s instructions, for BioFACT™ Taq DNA Polymerase (BioFACT Co., Ltd., Daejeon, Republic of Korea). We purified the PCR product using a FavorPrep™ GEL/PCR purification kit (Favorgen Biotech Corp., Kaohsiung, Taiwan), and then sequenced it with an ABI PRISM 3730XL analyzer (ABI, Foster City, CA, USA). The sequencing results for each sample were analyzed using Finch TV software version 1.4 (Geospiza Inc., Seattle, WA, USA).

### 2.3. Statistical Analysis

To assess whether the sample selection in the experiment was appropriate and whether the allele or genotype frequencies in the specific population remained consistent, we conducted a Hardy–Weinberg equilibrium (HWE) test using Michael H. Court’s calculator (Excel) [52,53]. A *p*-value of less than 0.05 indicates a deviation from HWE, suggesting that the observed genotype frequencies significantly differ from the expected distributions [53,54]. For the LD analysis, we examined the statistical relationships between SNPs at each gene locus. LD, which quantifies the correlation between two genetic loci, was evaluated using the r^2^ and D’ values [55]. High values indicate a strong linkage between loci, implying associated genetic regions, whereas low values suggest weak correlation. The LD and haplotype distributions were calculated using Haploview version 4.2 (Broad Institute, Cambridge, MA, USA).

### 2.4. In Silico Prediction of the Effects of Non-Synonymous SNPs in the Feline PRND Gene

We used four prediction tools to assess whether the substituted amino acids in non-synonymous SNPs affect protein structure or function. PolyPhen-2 evaluates the effects of non-synonymous SNPs on protein structure and function by analyzing the difference in PSIC (position-specific independent count) scores (http://genetics.bwh.harvard.edu/pph2/ (accessed on 10 May 2024)) [56]. The results are categorized as “probably damaging”, “possibly damaging”, or “benign” based on the associated risk level. SIFT assesses the influence of amino acid substitutions on protein function based on sequence homology, assuming that evolutionary conservation, as reflected in sequence alignment, is crucial for protein functionality (https://sift.bii.a-star.edu.sg/index.html (accessed on 10 May 2024)) [57]. Its scoring system ranges from 0 to 1, with values below 0.05 indicating deleterious effects. PANTHER utilizes PANTHER-PSEP (position-specific evolutionary preservation) to estimate the functional effect of non-synonymous SNPs (https://www.pantherdb.org/tools/csnpScoreForm.jsp? (accessed on 10 May 2024)) [58]. Positions exhibiting prolonged conservation are anticipated to have more severe effects. These effects are quantified as Pdel (probability of deleterious effect), with outcomes classified as “probably damaging”, “possibly damaging”, or “probably benign”. Missense3D predicts structural damage that may affect protein stability, indicating structural clashes with surrounding residues, the disruption of salt bridges, and alterations in the secondary structure (http://missense3d.bc.ic.ac.uk/missense3d/ (accessed on 10 May 2024)) [59]. According to the Missense3D criteria, a variant is flagged with a clash warning if the mutant structure has a MolProbity clash score greater than 30, for the clash score increases by more than 18 compared with the wild type.

### 2.5. Protein Structure Prediction of Feline Doppel

The structure modeling of cat Doppel was performed by ColabFold v1.5.5: AlphaFold2 using MMseqs2 [60]. ColabFold uses MSAs (multiple sequence alignments) generated by MMseqs2 to predict protein structures via methods combined in AlphaFold2 or RoseTTAFold. The 3D model of cat Doppel was analyzed using the feline *PRND* sequence obtained in this study. The protein structure was visualized using Swiss PDB Viewer 4.1 and Missense3D. Hydrogen bonds (H-bonds) were detected using a Swiss PDB Viewer when the distance between the donor and acceptor residues ranged from 2.35 to 3.2 Å.

### 2.6. Genetic Linkage Analysis of PRNP and PRND Polymorphisms in Cats

To examine the genetic linkage between polymorphisms in the *PRNP* and *PRND* genes, we performed an LD analysis involving these two loci. Initially, we obtained a dataset of *PRNP* genotypes from 210 cat samples reported previously [42]. We then preprocessed the data to align the *PRNP* genotypes with *PRND* genotypes from the same individuals. Out of the entire genotype dataset, 207 samples were successfully matched. These samples were used to calculate r^2^ and D’ values between SNPs in the *PRNP* and *PRND* genes using Haploview version 4.2.

## 3. Results

### 3.1. Investigation of PRND Polymorphisms in Cats

**Figure 1:**
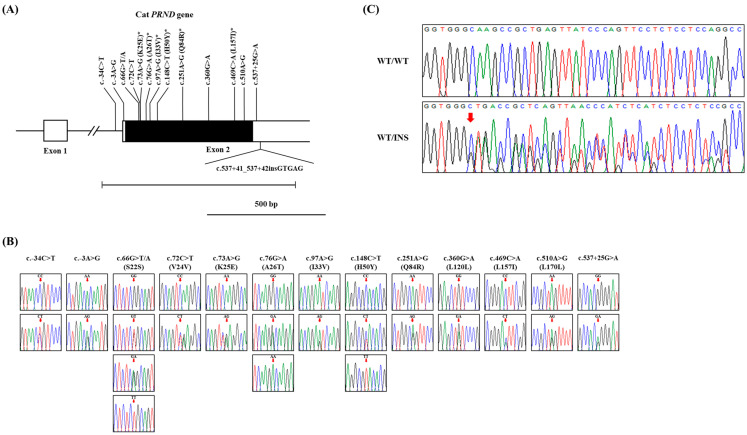
Identification of novel polymorphisms of the *prion-like* protein gene (*PRND*) in cats. (**A**) The gene diagram displays the genomic structure of the feline *PRND* gene. The open reading frame (ORF) within exon 2 is depicted as a black box, and the 5′ and 3′ untranslated regions (UTRs) from exon 1 to exon 2 are shown as white boxes. Horizontal lines with caps at the beginning and end indicate the full lengths of the PCR products analyzed in this study. Vertical and folded lines indicate the positions of the single nucleotide polymorphisms (SNPs) identified in this study, with an asterisk denoting each non-synonymous SNP. The Y-shaped bar indicates the insertion/deletion polymorphism. (**B**) Electropherograms display 13 novel SNPs discovered in the feline *PRND* gene. The genotypes for each SNP are shown: c.–34C>T, c.–3A>G, c.66G>T, c.72C>T, c.73A>G, c.76G>A, c.97A>G, c.148C>T, c.251A>G, c.360G>A, c.469C>A, c.510A>G, and c.537+25G>A. Arrows indicate the position of each SNP in the top label. (**C**) Electropherograms display the insertion polymorphism (c.537+41_537+42insGTGAG) discovered in the feline *PRND* gene. The upper panel shows the WT/WT homozygosity of the feline *PRND*. The lower panel shows WT/insertion heterozygosity of the feline *PRND*. Arrows indicate the start of the insertion. The peaks in the DNA sequence are color-coded, with green representing adenine, red for thymine, blue for cytosine, and black for guanine.

To identify *PRND* polymorphisms in cats, we analyzed DNA sequences targeting the ORF (537 bp) within exon 2 of the feline *PRND* gene. We found 13 novel SNPs: c.–34C>T in the intron region upstream of exon 2; c.–3A>G in the 5′ untranslated (UTR) region; c.66G>T/A, c.72C>T, c.73A>G, c.76G>A, c.97A>G, c.148C>T, c.251A>G, c.360G>A, c.469C>A, and c.510A>G in the ORF region; and c.537+25G>A in the 3′ UTR region (Figure 1A). Among those thirteen SNPs, the six at c.73A>G (K25E), c.76G>A (A26T), c.97A>G (I33V), c.148C>T (H50Y), c.251A>G (Q84R), and c.469C>A (L157I) are non-synonymous SNPs (Figure 1B). Interestingly, we observed two cases of heterozygosity for the c.66G>T/A SNP, which confirmed four genotypes (Figure 1B). In addition, we identified one insertion/deletion polymorphism (InDel), c.537+41_537+42insGTGAG, in the 3′ UTR region (Figure 1C). The detailed genotyping and allele frequencies of the 14 polymorphisms of the feline *PRND* gene are provided in Table 1.

We investigated the extent of LD among the 14 polymorphisms by calculating r^2^ and D’ values (Table 2). Among them, a strong LD (r^2^ > 0.3) was observed between c.–34C>T and c.537+25G>A; c.–3A>G, c.72C>T, and c.73A>G; c.97A>G and c.251A>G; and c.510A>G, c.537+25G>A, and c.537+41_537+42insGTGAG. In addition, most *PRND* SNPs showed strong LD with D’ values close to 1. However, certain SNPs pairs, such as c.76G>A and c.469C>A (0.027); c.360G>A and c.510A>G (0.085); c.469C>A and c.510A>G, c.537+25G>A, and c.537+41_537+42insGTGAG, displayed weak linkage, with D’ values close to 0. We also examined the haplotype frequencies of the *PRND* polymorphisms (Table 3). The most frequently observed haplotype was CAGCAGACAGCAGWt (55.2%), followed by CATCAGACAGCAGWt (23.8%), CAGCAAACAGCAGWt (7.7%), CAGCAGACAGCGGIns (2.4%), CAGCAGACAGAAGWt (2.2%), CGGTGGACAGCAGWt (1.9%), and CGGCAGGCGGCAGWt (1.7%). The detailed r^2^ and D’ values are provided in Table 2, while the haplotypes are presented in Table 3.

### 3.2. Predicting the Effects of Non-Synonymous SNPs on the Function and Properties of Feline Doppel

To estimate the extent to which non-synonymous SNPs in the feline *PRND* gene have damaging effects, we used in silico prediction tools, PolyPhen-2, SIFT, and PANTHER (Table 4). The PolyPhen-2 analysis predicted that the SNP c.76G>A (A26T) was “Possibly damaging” with a score of 0.686, whereas the other five non-synonymous SNPs were categorized as “Benign”. SIFT predicted that two non-synonymous SNPs, c.251A>G (Q84R) and c.469C>A (L157I), were damaging, with scores of 0.00. In contrast, PANTHER predicted that all of the non-synonymous SNPs would be benign. We also used Missense3D to check for any potentially harmful structural changes resulting from the amino acid substitutions (Table 4). Among the six non-synonymous SNPs, five showed no detectable structural damage. However, the SNP c.97A>G (I33V) exhibited a potential structural clash. The wildtype amino acid isoleucine at codon 33 had a clash score of 12.7, and the substituted amino acid valine increased the clash score to 35.26.

**Figure 2:**
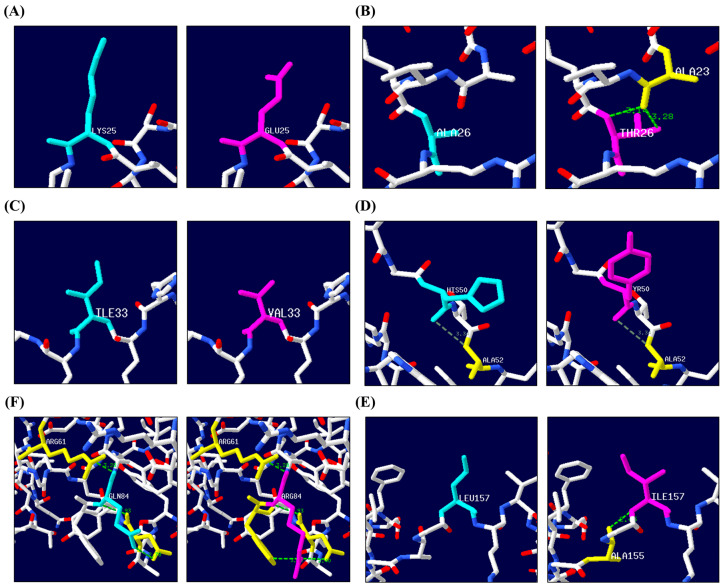
Structural analysis of the feline *prion-like* protein (Doppel) according to the non-synonymous single nucleotide polymorphisms (SNPs). The tertiary structures of cat Doppel with the SNPs (**A**) SNP c.73A>G (K25E), (**B**) SNP c.76G>A (A26T), (**C**) SNP c.97A>G (I33V), (**D**) SNP c.148C>T (H50Y), (**E**) SNP c.251A>G (Q84R), and (**F**) SNP c.469C>A (L157I) were predicted by ColabFold v1.5.5 and visualized using Swiss PDB Viewer 4.1. In each panel, the wildtype amino acids are highlighted in cyan on the left, and the substituted amino acids are shown in magenta on the right. Hydrogen bonds (H-bonds) within the specified distance range are depicted with light green dotted lines, and weaker hydrogen bonds, which can extend by an additional 0.05 Å by default, are shown in gray. The residues surrounding these hydrogen bonds are highlighted in yellow.

We used ColabFold to make 3D modeling predictions for the feline Doppel protein and thereby assess any structural changes caused by the non-synonymous SNPs. First, we used ColabFold to predict the protein structure from the amino acid sequence of the feline *PRND* gene. We then utilized the predicted 3D structure to analyze changes in H-bonds with surrounding amino acid residues caused by the amino acid substitutions resulting from the non-synonymous SNPs, using the Swiss PDB Viewer (Figure 2A–E, Supplementary Figure S1 and Table S1). For SNPs c.73A>G (K25E) and c.97A>G (I33V), no H-bonds with surrounding amino acid residues were detected (Figure 2A,C). In SNP c.76G>A (A26T), the substitution of alanine to threonine formed two H-bonds with alanine at codon 23 at distances of 3.14 Å and 3.28 Å (Figure 2B). For SNP c.148C>T (H50Y), weak H-bonds were maintained with alanine at codon 52, despite the amino acid substitution (3.34 Å) (Figure 2D). In the case of SNP c.251A>G (Q84R), the wild-type amino acid glutamine formed three H-bonds: one with arginine at codon 61 (3.28 Å) and two with asparagine at codon 81 (2.93 Å and 2.78 Å). The substituted amino acid arginine, however, formed four H-bonds: one with arginine at codon 61 (3.28 Å), two with asparagine at codon 81 (2.93 Å and 2.40 Å), and one with tryptophan at codon 83 (3.20 Å) (Figure 2E). For SNP c.469C>A (L157I), the substitution of leucine to isoleucine resulted in an H-bond with alanine at codon 155 at a distance of 2.45 Å (Figure 2F). Additionally, we discovered that a 3D structure of feline *PRND*, predicted by AlphaFold, is already available on UniProt (M3VWQ4_FELCA).

### 3.3. Investigation of Genetic Linkage Between Feline PRNP and PRND SNPs

A previous study identified fourteen synonymous SNPs and one InDel in the feline *PRNP* gene [42]. To assess the strength of the genetic linkage between feline *PRND* and *PRNP* SNPs, we conducted an LD analysis using r^2^ and D’ values. LD scores were calculated for 207 cats, excluding 3 animals with mismatched genotyping data for the *PRNP* gene. The *PRND* SNP c.66G>T showed a strong linkage with the *PRNP* SNP c.201C>T, with an *r*^2^ value of 0.604. In addition, the *PRND* SNPs c.97A>G and c.251A>G were strongly linked with the *PRNP* InDel c.214_240delCCCCACGCCGGCGGAGGCTGGGGTCAG, with an *r*^2^ value of 0.873. The remaining linkages were weak, with *r*^2^ scores below 0.3. Based on D’ values, all *PRND* SNPs, except for *PRND* c. –34C>T, were strongly linked with the *PRNP* SNP c.201C>T. Additionally, most *PRND* SNPs exhibited strong linkage with the *PRNP* InDel c.214_240delCCCCACGCCGGCGGAGGCTGGGGTCAG. However, *PRND* c.72C>T, c.73A>G, and c.76G>A display weaker linkage, with D’ values below 0.25.

## 4. Discussion

Previous studies have reported that polymorphisms in the *PRND* gene are rare in prion disease-resistant species, such as dogs and horses, despite the gene being highly conserved across species. In contrast, prion disease-susceptible species, including humans, cattle, sheep, and goats, are highly polymorphic in the *PRND* gene [39,46,47,48,49,50,51,61,62]. Case–controlled studies have identified associations between *PRND* polymorphisms and prion disease susceptibility in humans at codon 174 and the 3′ untranslated region (UTR) +28 [36,46,47], in cattle at codons 95 and 132 [48], in sheep at codon 26 [49], and in goats at codon 10 [51]. Furthermore, significant correlations between *PRNP* and *PRND* gene polymorphisms have been observed in both sheep and goats [49,50]. These findings suggest that *PRND* polymorphisms may play an indirect yet crucial role in modulating prion disease susceptibility across various species.

In this study, we identified 13 novel SNPs, including six non-synonymous SNPs, and one InDel in the feline *PRND* gene (Figure 1 and Table 1), and analyzed the genetic characteristics of *PRND* polymorphism in cats. Our LD analysis with an r^2^ value between *PRND* SNPs revealed four pairs with strong LD, including the non-synonymous SNP pairs c.97A>G (I33V) and c.251A>G (Q84R) (Table 2). Overall, most *PRND* SNPs showed strong linkage based on D’ values, except for certain SNPs pairs, such as c.76G>A and c.469C>A; c.360G>A and c.510A>G; c.469C>A and c.510A>G, c.537+25G>A, and c.537+41_537+42insGTGAG. It is important to note that both r² and D’ values can be influenced by allele frequencies and the absence of certain haplotypes. In particular, rare alleles may show low r² values despite the presence of linkage, highlighting the importance of considering allele frequency in such analyses [63].

Additionally, we identified two major haplotypes, CAGCAGACAGCAGWt and CATCAGACAGCAGWt, while other haplotypes were observed at relatively low frequencies of less than 10% (Table 3). Interestingly, genotype frequencies for SNPs c. –3A>G and c.148C>T (H50Y) significantly deviated from HWE expectations (*p* < 0.05). These deviations may reflect differences in genotype distribution across cat breeds [42]. To address this, a comparative analysis with larger, breed-specific sample sizes would be beneficial for future studies. The findings of this study provide a foundational reference for such investigations. Cats are a primary host species for FSE, and the highly polymorphic nature we found in the feline *PRND* gene suggests that *PRND* polymorphism in cats share genetic characteristics with other prion disease-susceptible animals.

We investigated the impact of these non-synonymous SNPs on the feline Doppel protein, and four of them were predicted to have deleterious effects (Table 4). SNP c.97A>G (I33V) was predicted by Missense3D to introduce structural clashes into the Doppel protein. These clashes were analyzed with a focus on the localized effects of the amino acid substitution rather than the entire protein structure, which is an important consideration [59]. PolyPhen-2 and SIFT predicted that SNPs c.76G>A (A26T), c.251A>G (Q84R), and c.469C>A (L157I) were likely to have harmful effects on protein function and structure, respectively. Interestingly, these predictions are consistent with the increase in the number of H-bonds found after making the amino acid substitutions in the 3D models (Figure 2). Unfortunately, although an increase in H-bonds can suggest enhanced protein stability, we were unable to determine the connection between that outcome and the predicted harmful effects on the protein.

In this study, we utilized individual prediction tools for SNP analysis based on its unique predictive algorithm. Missense3D focuses on potential structural alterations caused by amino acid substitutions [59], while PANTHER employs evolutionary conservation data to estimate the biological impact of variations [58]. PolyPhen-2 applies sequence-based approaches, whereas SIFT emphasizes the evolutionary conservation and predicts functional impacts based on residue substitutions [56,57]. These diverse focal points lead to inherent inconsistencies in the results, as each tool assesses the substitutions from distinct biochemical or evolutionary perspectives. As shown in Table 2, SNP c.97A>G (I33V) showed strong LD with SNP c.251A>G (Q84R), with an r² value of 1; however, these SNPs did not consistently show deleterious effects across the same prediction tools. Based on these results, SNP c.97A>G (I33V) is thought to have a detrimental impact from a structural perspective, whereas SNP c.251A>G (Q84R) likely exerts harmful effects from an evolutionary perspective. This highlights how variations such as SNPs c.97A>G (I33V) and c.251A>G (Q84R) may yield inconsistent predictions due to methodological differences. Additionally, the prediction tools used in this study assess the impact of amino acid substitutions at single positions within the protein structure. We confirmed that SNPs c.97A>G (I33V) and c.251A>G (Q84R) exhibit a co-occurring distribution and explored methods for simultaneously introducing both SNPs. Since conventional in silico tools are limited to evaluating amino acid changes at multi codons, we conducted two analyses: (1) substituting c.251A>G (Q84R) into a sequence where c.97A>G (I33V) was preset as valine, and (2) substituting c.97A>G (I33V) into a sequence where c.251A>G (Q84R) was preset as arginine. When c.97A>G (I33V) was first substituted to valine, followed by the introduction of c.251A>G (Q84R), only the SIFT generated a deleterious outcome. As shown in Table 4, this result aligns with the individual predictions; however, no synergistic interaction between c.97A>G (I33V) and c.251A>G (Q84R) was observed. The development of algorithms capable of evaluating the effects of multiple codon substitutions within a single sequence would be beneficial for future studies. Such advancements could enable a deeper exploration of potential effects arising from simultaneous amino acid changes at multiple positions. Furthermore, future research should consider incorporating meta-predictor tools, such as MetaSNP, to facilitate a more comprehensive and integrated analysis. The Doppel protein is predominantly expressed in the testes, where it plays a key role in male fertility and reproductive functions [64,65]. Notably, previous studies in sheep, a primary host species for scrapie, have shown that sperm production capacity is associated with SNP c.78G>A (A26A) in the ovine *PRND* gene [49,66]. These findings suggest that *PRND* polymorphisms may play a critical role in influencing protein function, highlighting the need for further functional studies to explore the detailed relationship between these genetic variations and the role of the Doppel protein across various species.

Our LD analysis between the feline *PRND* and *PRNP* genes identified two pairs with strong linkage (Table 5). The *PRND* SNP c.66G>T and *PRNP* SNP c.201C>T showed a strong linkage (r² value of 0.604). However, because they are both synonymous SNPs, it is challenging to extend this finding to functional relevance. In contrast, the *PRND* SNPs c.97A>G and c.251A>G showed a strong genetic association with the *PRNP* InDel c.214_240delCCCCACGCCGGCGGAGGCTGGGGTCAG (p.76_84del PHAGGGWGQ), with an r² value of 0.873. All of those variants are non-synonymous SNPs. Notably, the *PRNP* InDel is located within the nonapeptide repeat R3 region, which is part of the tandem repeat region in the PrP protein, and is believed to be associated with the progression rate of prion diseases [42,67]. The strong LD between *PRND* and *PRNP* in this region suggests that *PRND* polymorphisms may indirectly influence the pathogenicity of prion diseases by altering the structure of *PRNP*. Interestingly, all three polymorphisms were predicted to have detrimental effects on protein structure and function, although those results were measured using different prediction tools (Table 4) [42]. Previous studies have reported weak genetic linkage between the *PRND* and *PRNP* genes in prion disease-resistant species, but stronger linkage in prion disease-susceptible species [41,61]. Our findings deepen our understanding of the role of *PRND* polymorphisms in prion disease susceptibility by means of a genetic linkage between these two genes.

## 5. Conclusions

We identified thirteen novel SNPs and one InDel in the feline *PRND* gene. Among the six non-synonymous SNPs, our analysis predicted that four, c.76G>A (A26T), c.97A>G (I33V), c.251A>G (Q84R), and c.469C>A (L157I), might have detrimental effects on the structure and function of the Doppel protein. Additionally, we observed strong linkage between two *PRND* SNPs, c.97A>G and c.251A>G, and the *PRNP* InDel c.214_240del within the nonapeptide repeat region, which suggests a potential association with prion disease susceptibility. These findings underscore the need for further functional studies to elucidate the biological significance of these genetic variations and their potential contributions to the mechanisms of prion disease progression in felines.

## Figures and Tables

**Table 1:** Genotype and allele frequencies of polymorphisms of the *prion-like* protein gene (*PRND)* in cats.

Polymorphisms	Genotype Frequency, *n* (%)				Allele Frequency, *n* (%)			HWE
c.–34C>T	CC	CT	TT		C	T		
	206	4	0		416	4		0.89
	(98.1)	(1.9)	(0)		(99.05)	(0.95)		
c.–3A>G	AA	AG	GG		A	G		
	188	19	3		395	25		0.01
	(89.52)	(9.05)	(1.43)		(94.05)	(5.95)		
c.66G>T/A	GG	GT	GA	TT	G	T	A	
(S22S)	122	73	1	14	317	101	1	0.5 *
	(58.1)	(34.76)	(0.48)	(6.67)	(75.71)	(24.05)	(0.24)	0.96 **
c.72C>T	CC	CT	TT		C	T		
(V24V)	201	9	0		411	9		0.75
	(95.71)	(4.29)	(0)		(97.86)	(2.14)		
c.73A>G	AA	AG	GG		A	G		
(K25E)	194	15	1		403	17		0.24
	(92.38)	(7.14)	(0.48)		(95.95)	(4.05)		
c.76G>A	GG	GA	AA		G	A		
(A26T)	177	32	1		386	34		0.73
	(84.29)	(15.24)	(0.48)		(91.9)	(8.10)		
c.97A>G	AA	AG	GG		A	G		
(I33V)	203	7	0		413	7		0.81
	(96.67)	(3.33)	(0)		(98.33)	(1.67)		
c.148C>T	CC	CT	TT		C	T		
(H50Y)	206	3	1		415	5		0.00
	(98.1)	(1.43)	(0.48)		(98.81)	(1.19)		
c.251A>G	AA	AG	GG		A	G		
(Q84R)	203	7	0		413	7		0.81
	(96.67)	(3.33)	(0)		(98.33)	(1.67)		
c.360G>A	GG	GA	AA		G	A		
(L120L)	204	6	0		414	6		0.83
	(97.14)	(2.86)	(0)		(98.57)	(1.43)		
c.469C>A	CC	CA	AA		C	A		
(L157I)	199	11	0		409	11		0.7
	(94.76)	(5.24)	(0)		(97.38)	(2.62)		
c.510A>G	AA	AG	GG		A	G		
(L170L)	191	19	0		401	19		0.49
	(90.95)	(9.05)	(0)		(95.48)	(4.52)		
c.537+25G>A	GG	GA	AA		G	A		
	202	8	0		412	8		0.78
	(96.19)	(3.81)	(0)		(98.1)	(1.9)		
c.537+41_537+42 insGTGAG	WT/WT	WT/INS	INS/INS		WT	INS		
	193	17	0		403	17		0.54
	(91.9)	(8.1)	(0)		(95.95)	(4.05)		

* This value represents the Hardy–Weinberg equilibrium (HWE) measure across GG homozygotes, GT heterozygotes, and TT homozygotes at SNP c.66G>T/A. ** This value represents the HWE measure across GG homozygotes and GA heterozygotes at SNP c.66G>T/A.

**Table 2:** The linkage disequilibrium (LD) of polymorphisms of the *prion-like* protein gene (*PRND)* in cats.

	*r* ^2^	c. –34C>T	c. –3A>G	c.66G>T	c.72C>T	c.73A>G	c.76G>A	c.97A>G	c.148C>T	c.251A>G	c.360G>A	c.469C>A	c.510A>G	c.537+25 G>A	c.537+41_537+42 insGTGAG
D’															
c.–34C>T		-	0.001	0.003	0	0	0.001	0	0	0	0	0	0.203	**0.495**	0.228
c.–3A>G		1	-	0.02	**0.346**	**0.666**	0.006	0.268	0.116	0.268	0.229	0.002	0.003	0.001	0.003
c.66G>T		1	1	-	0.007	0.014	0.017	0.005	0.004	0.005	0.005	0.009	0.015	0.006	0.014
c.72C>T		1	1	1	-	**0.519**	0.002	0.051	0	0.051	0.011	0.001	0.001	0	0.001
c.73A>G		1	1	1	1	-	0.004	0.02	0.177	0.02	0.231	0.001	0.001	0.001	0.002
c.76G>A		1	1	0.785	1	1	-	0	0.001	0	0.001	0	0.004	0	0.004
c.97A>G		1	1	1	0.257	0.226	0.273	-	0	**1**	0	0	0.001	0	0.001
c.148C>T		1	0.781	1	1	0.778	1	1	-	0	0	0	0.001	0	0.001
c.251A>G		1	1	1	0.257	0.226	0.273	1	1	-	0	0	0.001	0	0.001
c.360G>A		1	1	1	0.131	0.821	1	1	1	1	-	0	0.002	0	0.001
c.469C>A		1	1	1	1	1	0.027	1	1	1	1	-	0	0.004	0
c.510A>G		1	1	1	1	0.779	1	1	1	1	0.085	0	-	**0.307**	**0.89**
c.537+25 G>A		1	1	1	1	1	0.521	1	1	1	1	0.078	0.865	-	**0.345**
c.537+41_ 537+42ins GTGAG		1	1	1	1	1	1	1	1	1	1	0.011	1	0.866	-

Bold texts indicates strong LD with r^2^>0.3.

**Table 3:** Haplotype frequencies of polymorphisms of the *prion-like* protein gene (*PRND)* in cats.

	c.–34C>T	c.–3A>G	c.66G>T	c.72C>T	c.73A>G	c.76G>A	c.97A>G	c.148C>T	c.251A>G	c.360G>A	c.469C>A	c.510A>G	c.537+25 G>A	c.537+41_537+42insGTGAG	n (%)
ht1	C	A	G	C	A	G	A	C	A	G	C	A	G	Wt	231 (55)
ht2	C	A	T	C	A	G	A	C	A	G	C	A	G	Wt	99 (23.6)
ht3	C	A	G	C	A	A	A	C	A	G	C	A	G	Wt	32 (7.6)
ht4	C	A	G	C	A	G	A	C	A	G	C	G	G	Ins	10 (2.4)
ht5	C	A	G	C	A	G	A	C	A	G	A	A	G	Wt	9 (2.1)
ht6	C	G	G	T	G	G	A	C	A	G	C	A	G	Wt	8 (1.9)
ht7	C	G	G	C	A	G	G	C	G	G	C	A	G	Wt	7 (1.7)
ht8	C	G	G	C	G	G	A	T	A	G	C	A	G	Wt	4 (1)
ht9	T	A	G	C	A	G	A	C	A	G	C	G	A	Ins	4 (1)
ht10	C	G	G	C	G	G	A	C	A	A	C	A	G	Wt	3 (0.7)
ht11	C	A	G	C	A	G	A	C	A	G	C	G	A	Ins	1 (0.2)
Other *	C	A	G	C	A	A	A	C	A	G	A	A	G	Wt	12 (2.8)

* Other contains rare haplotypes with a frequency of <0.2%.

**Table 4:** In silico prediction of the functional effects of non-synonymous single nucleotide polymorphisms (SNPs) of the *prion-like* protein gene (*PRND*) in cats.

Variations	PolyPhen-2		SIFT		PANTHER		Missense3D	
	Score	Prediction	Score	Prediction	Score	Prediction	Score	Prediction
c.73A>G (K25E)	0.001	Benign	0.17	Tolerated	0.27	Probably benign	-	No structural damage detected
c.76G>A (A26T)	0.686	Possibly damaging	0.56	Tolerated	0.27	Probably benign	-	No structural damage detected
c.97A>G (I33V)	0.082	Benign	0.48	Tolerated	0.27	Probably benign	35.26	Clash
c.148C>T (H50Y)	0.295	Benign	0.22	Tolerated	-	Not scored	-	No structural damage detected
c.251A>G (Q84R)	0.003	Benign	0	Damaging	0.27	Probably benign	-	No structural damage detected
c.469C>A (L157I)	0.284	Benign	0	Damaging	0.27	Probably benign	-	No structural damage detected

**Table 5:** The linkage disequilibrium (LD) between polymorphisms in the prion protein gene (*PRNP)* and the *prion-like* protein gene (*PRND)* in cats.

	*PRNP* c.201 C>T		*PRNP* c.214_240 delCCCCACGCCGGCGGAGGCTGGGGTCAG	
*PRND*	r^2^	D’	r^2^	D’
c.–34C>T	0	0.276	0	1
c.–3A>G	0.034	1	0.228	0.862
c.66G>T	**0.604**	1	0.006	1
c.72C>T	0.012	1	0.043	0.219
c.73A>G	0.023	1	0.016	0.186
c.76G>A	0.028	0.777	0.002	0.105
c.97A>G	0.009	1	**0.873**	1
c.148C>T	0.006	1	0	1
c.251A>G	0.009	1	**0.873**	1
c.360G>A	0.008	1	0	1
c.469C>A	0.014	1	0.001	1
c.510A>G	0.015	0.802	0.001	1
c.537+25G>A	0.003	0.545	0	1
c.537+41_537+42insGTGAG	0.013	0.77	0.001	1

Bold texts indicates strong LD with r^2^ > 0.3.

## Data Availability

All data generated or analyzed during this study are available from the corresponding author upon reasonable request.

## Supplementary Materials

The following supporting information can be downloaded at: https://www.mdpi.com/article/10.3390/ani14233438/s1, Table S1. Per-residue confidence score (pLDDT) for each amino acid in the predicted 3D model of cat Doppel protein. Figure S1: 3D structure of the full-length feline *prion-like* protein (Doppel). The SNPs identified in this study are mapped onto the 3D structure with the following color scheme: c.66G>T/A in purple, c.72C>T in green, c.73A>G in pink, c.76G>A in red, c.97A>G in yellow, c.148C>T in cyan, c.251A>G in brown, c.360G>A in magenta, c.469C>A in blue, and c.510A>G in orange.
