# Supplementary material for: First Report of Polymorphisms and Genetic Characteristics of Prion-like Protein Gene (PRND) in Cats
Source: Animals (Basel). 2024 Nov 27;14(23):3438. doi: 10.3390/ani14233438 (PMC11639842; doi:10.3390/ani14233438)
Supplement: Supplementary file 1 [file animals-14-03438-s001.zip › animals-3281610-supplementary/animals-3281610-supplementary/Supplementary table 1 v7.3.4.pdf]

Supplementary Table S1. Per-residue confidence score (pLDDT) for each amino acid in the predicted 3D model of cat Doppel protein

codon	Amino acid	value
1	MET	37.31
2	ARG	44.56
3	LYS	51.94
4	HIS	56.38
5	LEU	56
6	GLY	54.16
7	GLY	58.25
8	CYS	63.22
9	TRP	70.81
10	LEU	70.62
11	ALA	70.75
12	ILE	76.56
13	VAL	76
14	CYS	73.12
15	VAL	76.19
16	LEU	75.12
17	LEU	71.5
18	PHE	65.31
19	SER	68.88
20	GLN	58.78
21	LEU	56.69
22	SER	54.81
23	ALA	53.69
24	VAL	52.19
25	LYS	47.38
26	ALA	48.06
27	ARG	37.25
28	GLY	41
29	ILE	44.91
30	LYS	42.97
31	HIS	48.25
32	ARG	40.31
33	ILE	49.91
34	LYS	43.19
35	TRP	38.66
36	ASN	42

37	ARG	43.41
38	LYS	39.19
39	THR	41.47
40	LEU	42.25
41	PRO	42.5
42	SER	40.75
43	ILE	40.19
44	SER	44.34
45	GLN	40.09
46	VAL	40.56
47	THR	48.25
48	GLU	47.62
49	ALA	48.47
50	HIS	53.19
51	THR	48.62
52	ALA	52
53	GLU	51.72
54	ILE	51.19
55	ARG	52.03
56	PRO	49.34
57	GLY	52.19
58	ALA	63.56
59	PHE	72.81
60	ILE	71.06
61	ARG	77.75
62	GLN	75.44
63	GLY	73.81
64	ARG	81.12
65	LYS	86
66	LEU	86.19
67	ASP	88.88
68	ILE	87.81
69	ASP	91.06
70	LEU	90.94
71	GLY	92.12
72	ALA	91.88
73	GLU	93.12
74	GLY	92.88
75	ASN	94.19
76	ARG	94.12
77	TYR	92.56

78	TYR	92.06
79	GLU	91.56
80	ALA	91.69
81	ASN	90
82	TYR	88.56
83	TRP	85.5
84	GLN	85.69
85	PHE	87
86	PRO	86.75
87	ASP	84.19
88	GLY	85.38
89	ILE	87.38
90	HIS	86.19
91	TYR	85.56
92	ASN	81.44
93	GLY	72.81
94	CYS	71.44
95	SER	65.12
96	GLU	64.25
97	ALA	64.38
98	ASN	68.81
99	VAL	74.25
100	THR	83.19
101	LYS	85.44
102	GLU	87.69
103	LYS	86.56
104	PHE	87.5
105	VAL	90.56
106	THR	91.69
107	SER	89.38
108	CYS	90.56
109	ILE	92.56
110	ASN	92.88
111	ALA	90
112	THR	89.62
113	GLN	89.44
114	VAL	89.12
115	ALA	86.56
116	ASN	85.38
117	GLN	84.06
118	GLU	80.81

119	GLU	78
120	LEU	79.81
121	SER	77.88
122	ARG	73.38
123	GLU	67.38
124	LYS	63.22
125	GLN	65.44
126	ASP	72
127	ASP	86.69
128	LYS	88.94
129	LEU	90.56
130	TYR	89.81
131	GLN	90.62
132	ARG	92.25
133	VAL	92.38
134	LEU	91.75
135	TRP	92.69
136	ARG	93.19
137	LEU	91.62
138	ILE	92.19
139	ARG	93.19
140	GLU	91.56
141	LEU	89.44
142	CYS	90.31
143	SER	89.81
144	VAL	86.56
145	LYS	83.31
146	HIS	74.12
147	CYS	71.19
148	ASP	67.38
149	PHE	60.09
150	TRP	55.78
151	LEU	44.84
152	GLU	44.59
153	ARG	40.44
154	GLY	38.88
155	ALA	42.88
156	GLY	35.75
157	LEU	40.66
158	ARG	41.03
159	VAL	37.5

160	THR	40.53
161	LEU	40.53
162	ASP	46.06
163	GLN	51.69
164	PRO	49.03
165	VAL	55.12
166	MET	56
167	LEU	59.88
168	CYS	60.56
169	PRO	63.62
170	LEU	70.38
171	VAL	71
172	PHE	68.81
173	ILE	76.19
174	TRP	72.94
175	PHE	64
176	ILE	71.62
177	VAL	72.44
178	THR	62.72
