# Supplementary figures and images for: First Report of Polymorphisms and Genetic Characteristics of Prion-like Protein Gene (PRND) in Cats
Source: Animals (Basel). 2024 Nov 27;14(23):3438. doi: 10.3390/ani14233438 (PMC11639842; doi:10.3390/ani14233438)

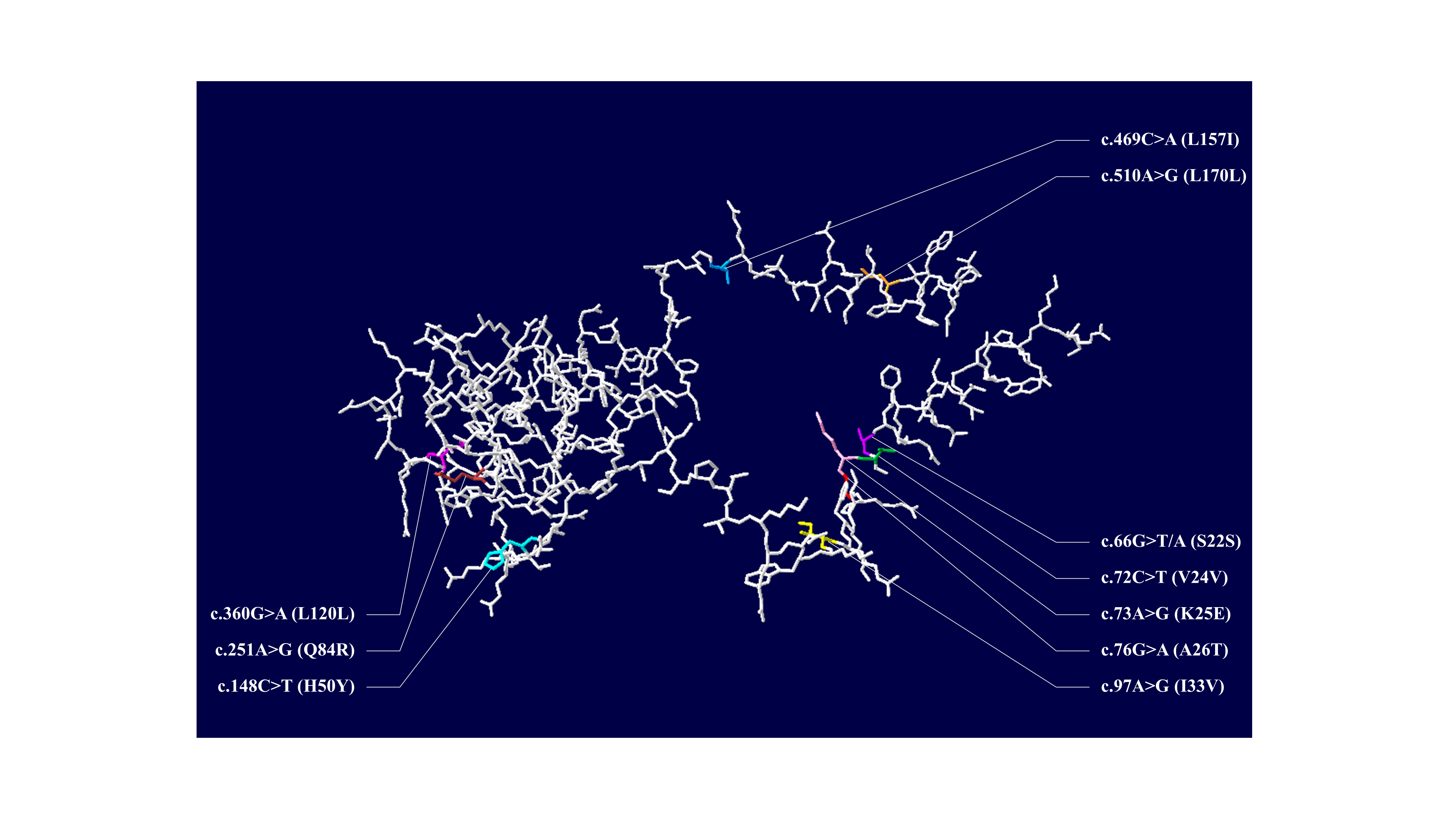

Supplement: Supplementary file 1 [file animals-14-03438-s001.zip › animals-3281610-supplementary/animals-3281610-supplementary/Supplementary Figure 1.TIF]
